# 6-Shogaol Induces Apoptosis in Human Hepatocellular Carcinoma Cells and Exhibits Anti-Tumor Activity *In Vivo* through Endoplasmic Reticulum Stress

**DOI:** 10.1371/journal.pone.0039664

**Published:** 2012-06-29

**Authors:** Rong Hu, Ping Zhou, Yong-Bo Peng, Xiaojun Xu, Jiang Ma, Qun Liu, Lei Zhang, Xiao-Dong Wen, Lian-Wen Qi, Ning Gao, Ping Li

**Affiliations:** 1 State Key Laboratory of Natural Medicines, China Pharmaceutical University, Nanjing, China; 2 Department of Pharmacognosy, College of Pharmacy, 3rd Military Medical University, Chongqing, China; German Cancer Research Center, Germany

## Abstract

6-Shogaol is an active compound isolated from Ginger (*Zingiber officinale* Rosc). In this work, we demonstrated that 6-shogaol induces apoptosis in human hepatocellular carcinoma cells in relation to caspase activation and endoplasmic reticulum (ER) stress signaling. Proteomic analysis revealed that ER stress was accompanied by 6-shogaol-induced apoptosis in hepatocellular carcinoma cells. 6-shogaol affected the ER stress signaling by regulating unfolded protein response (UPR) sensor PERK and its downstream target eIF2α. However, the effect on the other two UPR sensors IRE1 and ATF6 was not obvious. In prolonged ER stress, 6-shogaol inhibited the phosphorylation of eIF2α and triggered apoptosis in SMMC-7721 cells. Salubrinal, an activator of the PERK/eIF2α pathway, strikingly enhanced the phosphorylation of eIF2α in SMMC-7721 cells with no toxicity. However, combined treatment with 6-shogaol and salubrinal resulted in significantly increase of apoptosis and dephosphorylation of eIF2α. Overexpression of eIF2α prevented 6-shogaol-mediated apoptosis in SMMC-7721 cells, whereas inhibition of eIF2α by small interfering RNA markedly enhanced 6-shogaol-mediated cell death. Furthermore, 6-shogaol-mediated inhibition of tumor growth of mouse SMMC-7721 xenograft was associated with induction of apoptosis, activation of caspase-3, and inactivation of eIF2α. Altogether our results indicate that the PERK/eIF2α pathway plays an important role in 6-shogaol-mediated ER stress and apoptosis in SMMC-7721 cells *in vitro* and *in vivo*.

## Introduction

**Figure 1:**
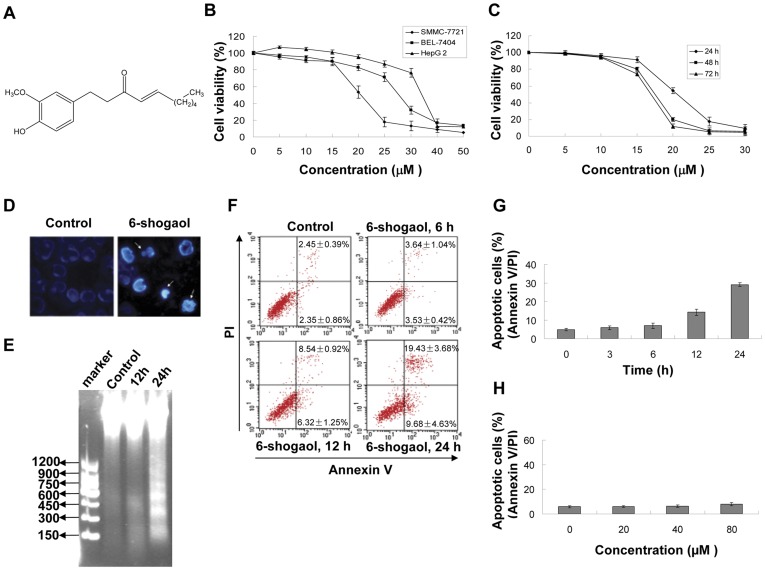
Effect of 6-shogaol on viability and apoptosis in HCC cells. (A) The chemical structure of 6-shogaol. (B) SMMC-7721, BEL-7404 and HepG2 cells were treated with 5, 10, 15, 20, 25, 30, 40 and 50 µM 6-shogaol for 24 h respectively, and the cell viability was determined by MTT assay. (C) SMMC-7721 cells were treated with 5, 10, 15, 20, 25 and 30 µM 6-shogaol for 24, 48, and 72 h respectively, and the cell viability was examined by MTT assay. (D) Cells (1×10^6^) were treated with 20 µM 6-shogaol for 24 h and stained with Hoechst 33258. Original magnification ×400. (E) Cells were treated with 20 µM 6-shogoal for 12 and 24 h. DNA frangemntation was determined. (F and G) SMMC-7721 cells were treated with 20 µM 6-shogaol for 3, 6, 12 and 24 h. The cells were stained with Annexin V/PI, and apoptosis was determined using flow cytometry. (H) Normal human liver HL-7702 cells were treated with 20, 40, and 80 µM 6-shogaol for 24 h. Apoptosis was determined using flow cytometry.

Ginger, derived from the rhizome of *Zingiber officinale* Rosc, is one of the most widely used spices around the world. It has been used as a common condiment in foods and beverages more than 2500 years [1]. In recent years, Ginger has received extensive attention as a botanical dietary supplement in the USA and Europe because of its anti-inflammatory, anti-oxidative and anti-tumor activities [1], [2]. 6-Shogaol (Figure 1A), the dehydration products of 6-gingerol, extracted from Ginger, exhibits much stronger anti-tumor activity than 6-gingerol [3]. In recent studies, 6-shogaol was reported to exhibit anti-tumor activity in various tumor cell lines [4]–[8]. However, detailed anti-tumor molecular mechanism of 6-shogaol in human hepatocellular carcinoma (HCC) cells still remains unclear.

Apoptosis is defined as a programmed cell death and has been proposed as an efficient anti-tumor mechanism. Malignant tumor cells can be eliminated after treatment with anticancer chemotherapies though apoptosis [9]. Recent studies suggested that apoptosis is coupled with ER stress [10], [11]. ER serves as a central organelle engaged in regulating protein synthesis, protein folding and intracellular calcium level, failure of which will cause ER stress [12], [13]. ER stress triggers signaling pathway termed as “unfolded protein response” (UPR) and leads to apoptosis if the ER stress becomes prolonged and severe [14]. The UPR is primarily regulated by three ER proximal sensors: PKR-like ER-associated kinase (PERK), activating transcription factor 6 (ATF6), and inositol requiring enzyme-1 (IRE1) [15]. During ER stress, PERK is dissociated from GRP78/BiP and turns to its phosphorylated form and then initiates the phosphorylation of eIF2α [16], [17]. eIF2α phosphorylation is required for cell survival by limiting the protein-folding load to prevent accumulation of misfolded proteins [18], and the subsequent additional stress [19]–[21]. In the absence of eIF2α phosphorylation, cells exhibit a higher rate of protein synthesis thus the demand for protein folding will increase. This includes increased pro-insulin folding and misfolding, and the later leads to accumulation of misfolded protein in the ER, thereby enhanced cell death [22]–[24].

In this work, a comparative proteomics approach was used to identify proteins alteration and explore the possible molecular basis of 6-shogaol-induced apoptosis in SMMC-7721 cells. The differentially expressed proteins were identified by the two-dimensional gel electrophoresis (2-DE) and LC-MS/MS. The UPR related proteins were further confirmed by western blot analysis. Through pharmacologic and genetic approaches, we demonstrated that the inhibition of eIF2α phosphorylation plays a pivotal role in 6-shogaol induced ER stress and apoptosis in SMMC-7721 cells *in vitro* and *in vivo*.

## Materials and Methods

### Cell Lines and Chemicals

HepG2 cells were purchased from American Type Culture Collection (Manassas, VA, USA). SMMC-7721, BEL-7404 and HL-7702 cells were purchased from Cell Bank of Chinese Academy of Sciences, Shanghai. 6-Shogaol was separated from ginger supercritical fluid extraction. Salubrinal was purchased from Alexis (Carlsbad, CA, USA). Antibodies against GRP78, GADD153, GRP94, PERK, eIF2α, phospho-eIF2α, PARP, HSP70, caspase-3, IRE1 were purchased from Cell Signaling Technology (Beverly, MA); Anti-β-actin and phospho-PERK antibodies were from Santa Cruz Biotechnology (Santa Cruz, CA, USA); Anti-ATF6 antibody was from Millipore (Billerica, MA, USA) and antibodies against phospho-IRE1, Calpain-1, Calpain-2 were from Abcam (Cambridge, MA, USA). All the other chemicals were purchased from either Amersham Biosciences or Sigma-Aldrich.

### Apoptosis Assay

The following methods were used to assess drug-induced apoptotic cell death: The cytotoxicity of 6-shogaol was evaluated by MTT assay. Morphological changes of apoptosis were stained with Hoechst 33258 and detected by fluorescence microscopy. The apoptotic cells were determined by flow cytometry as described before [25]. DNA laddering was practiced according to Genomic DNA Purificaion Kit (Promega, WI, USA). DNA degradation was detected by flow cytometric analysis.

### Caspase 3 Activity Assay

The activity of caspase-3 assay was carried out according to Caspase-3 Activity Assay Kit (Beyotime, Haimen, China). The caspase-3 activity assay is based on spectrophotometric detection of the chromophore p-nitronanilide (p-NA), after its cleavage from the labeled substrate, acetyl-Asp-Glu-Val-Asp p-nitroanilide (Ac-DEVD-pNA). Briey, SMMC-7721 cells were lysed after treatment with 6-shogaol (20 µM). Assays were performed on 96-well plates by incubating 30 µg total protein of cell lysate per sample and add reaction buffer (1% NP-40, 20 mM Tris–HCl (pH 7.5), 137 mM Nad, and 10% glycerol) to a total volume of 90 µL, containing 10 µL caspase-3 substrate (Ac-DEVD-pNA) (2 mM). Lysates were incubated at 37°C for 4 h. Samples were measured with an enzyme-linked immune-sorbent assay (ELISA) reader at an absorbance of 405 nm and the caspase activities were expressed as the percentage of enzyme activity compared with the control group. All the experiments were performed in triplicate.

### Two-dimensional Gel Electrophoresis (2-DE)

The total cellular proteins (300 µg) were diluted to 350 µL in hydration solution (8 M urea, 2 M thiourea, 4% CHAPS, 20 mM tris, 65 mM DTT and 0.5% IPG buffer), and then loaded on the Ready-Strip IPG strips (18 cm, pH 3–10, Nonlinear, Bio-Rad) for IEF. The strips were placed into Amersham Biosciences IPGphor IEF (Amersham) and were rehydrated and then separated based on p*I*. After IEF, the IPG strips were equilibrated for 15 min in a buffer containing 50 mM Tris-HCl, pH 6.8, 30% glycerol, 6 M urea, 1% SDS, and 0.2% DTT, followed by further treatment to prevent reformation of disulfide bonds in a similar buffer (but containing 3% iodoacetamide instead of DTT) for 15 min and then directly applied onto 12.5% homogeneous SDS-PAGE gels for electrophoresis using a Protean II xi cell system (Bio-Rad, USA). The gels were then stained with coomassie brilliant blue and analyzed with the Image-Master 2D Platinum software (GE Healthcare). For each experiment, the gels were run in triplicate.

### Protein Spot In-gel Enzymatic Digestion and LC-ESI-MS/MS Identification

The protein spots of interest were excised, distained and digested with trypsin (Promega, WI, USA). The supernatant peptide were sequentially extracted with peptide extraction solution (5% formic acid (FA), 67% ACN) at 37°C for 30 min, and then dissolved in sample buffer (2% ACN in water with 0.1% FA) after drying and subjected to liquid chromatography (LC Packings, Dionex, Sunnyvale, CA, USA) coupled with electrospray ionisation tandem mass spectrometry (Esquire HCT, Bruker, Germany). Data-dependent acquisition was controlled by chromeleon software (Dionex, Sunnyvale, CA, USA). The MS/MS data was input to MASCOT 2.0 program (MatrixScience, Boston, MA, USA) against IPI database v3.26 to identify the protein.

### Stable eIF2α Over-expression and Knockdown Cell Lines

eIF2α cDNA was amplified by RT-PCR and cloned into the lentivirus vector pWPI-Rho-GDI2 to obtain pWPI-Rho-GDI2-eIF2α. eIF2α shRNA sequence was designed and double strand oligonucleotides were synthesized and cloned onto the pLKO.1-SH plasmid to obtain pLKO.1-SH-shRNA/eIF2α. Then the plasmids were transformed into E. coli DH5α. Purified plasmids form the positive clones confirmed by PCR and sequencing. 239T cell lines were transfected with pWPI-Rho-GDI2, psPAX2, pMD and pLKO.1-SH, psPAX2, pMD respectively to produce lentivirus. The supernatant with lentivirus were collected and centrifuged for 72 h to get positive lentivirus. The virus titer was determined by real time PCR. SMMC-7721 cells were transduced with lentivirus and selected for stable expression of the trans-gene by the addition of 3 µg/mL puromycin.

### Western Blot Analysis

After treatment, total cellular samples were washed twice with cold PBS and lysed in RIPA lysis buffer. The total cellular protein extracts were separated by SDS-PAGE, and transferred to nitrocellulose membrane. Membranes were blocked with 5% fat-free dry milk in 1× TBS containing 0.05% Tween 20 under room temperate for 2 h and incubated with primary antibodies at 4°C overnight. Protein bands were detected by incubation with horseradish peroxidase-conjugated antibodies, and visualized with enhanced chemiluminescence reagent (Amersham, USA) as reported [26].

### Xenograft Assay

All animal studies were conducted according to protocols approved by the Institutional Animal Care and Use Committee (IACUC) of the China Pharmaceutical University (No. 20110302-rat 32). Male SCID mice were purchased from Charles River Laboratories (Wilmington, MA). A total of 3×10^6^ SMMC-7721 cells were inoculated subcutaneously into SCID mice. When the tumors reached an average volume of about 0.1 cm^3^, the mice were randomly divided into control and treatment groups (n = 8 animals per group). For the treatment groups, the mice were administered with 6-shogaol (10 mg/kg or 50 mg/kg, i.p. for 28 days). The control group received the vehicle buffer alone. Tumor size and body weight were measured after treatment at various time intervals throughout the study. Tumor volumes were calculated by the formula: length × width^2^ ×0.52 in millimeters. At the termination of the experiment, mice were sacrificed at 24 h after the last administration of compound. Tumor samples were excised and weighed, then fixed in paraformaldehyde. Paraffin-embedded tissues were sectioned and processed for H&E, TUNEL and immunohistochemical staining.

### TUNEL Assay

The apoptotic cells in tissue samples were detected using an In Situ Cell Death Detection kit (Roche, Mannheim, Germany) according to the manufacturer’s manual. After deparaffinization and permeabilization, tissue sections were incubated with proteinase K at room temperature for 15 min. The sections were then incubated with the TUNEL reaction mixture that contains terminal deoxynucleotidyl transferase (TdT) and fluorescein-dUTP at 37°C for 1 h. After three times wash with PBS, the sections were incubated with the Converter-POD which contains anti-fluorescein antibody conjugated with horse-radish peroxidase (POD) at room temperature for 30 minutes. Again, after three times wash with PBS, the sections were incubated with 0.05% 3-3'-diaminobenzidine tetrahydrochloride (DAB) and analyzed under light microscope.

### Histological and Immunohistochemical Evaluation

At the termination of xenograft experiments, tumor tissues from representative mice were sectioned, embedded in paraffin, and stained with hematoxylin and eosin for histopathologic evaluation. For immunohistochemical analysis, tissue sections 4 µm in thickness were de-waxed and rehydrated in xylene and graded alcohols. Antigen retrieval was performed with 0.01 M citrate buffer at pH 6.0 in a 95°C water bath for 20 min. Slides were allowed to cool for another 20 min, followed by sequential rinsing in PBS and TBS-T buffer. Endogenous peroxidase activity was quenched by incubation in TBS-T containing 3% hydrogen peroxide. Each incubation step was carried out at room temperature and was followed by three sequential washes (5 min each) in TBS-T. After blocking with 10% goat serum for 1 h, sections were incubated with primary antibodies, washed three times in PBS, incubated with biotinylated secondary antibody for 1 h, followed by incubation with a streptavidin-peroxidase complex for another 1 h. After three additional washes in PBS, diaminobenzidine working solution was applied. Finally, the slides were counterstained in hematoxylin.

### Statistical Analysis

Data are presented as mean ± SE for the indicated number of independent experiments. Statistical differences between groups were calculated using Student’s two-tailed *t*-test. Differences were considered statistically significant for values *p*<0.05 or *p*<0.01.

## Results

### 6-Shogaol-induced Apoptosis in HCC Cells in Dose- and Time-dependent Manners

To investigate the cytotoxicity of 6-shogaol, several HCC cell lines were incubated with various concentrations of 6-shogaol for 24 h, and the cell viability was examined by MTT assay. 6-Shogaol exhibited cytotoxicity in all three HCC cells, SMMC-7721 was the most susceptive one (Figure 1B). As shown in Figure 1C, exposure of SMMC-7721 cells to 6-shogaol resulted in viability decrease in a time-dependent manner. By Hoechst 33258 staining, 6-shogaol treated cells showed apoptotic phenotypes, with signs of significant chromatin condensation, cell and nuclear shrinkage (Figure 1D and Figure S1D). DNA laddering analysis revealed that exposure of SMMC-7721 cells to 20 µM 6-shogaol for 12 h resulted in a slight increase in cellular DNA degradation and it became apparent after 24 h of drug exposure (Figure 1E), the DNA degradation analysis showed the same results (Figure S1A). Calpain-1 and Calpain-2 showed no significantly variation by Western blot (Figure S1B). In addition, flow cytometric analysis using Annexin V/PI staining further confirmed that cells exposed to 6-shogaol (20 µM) for 12 h resulted in a moderate increase in apoptosis, after 24 h of the drug adminstration it became obvious (Figure 1F, 1G and Figure S1C). In contrast, 6-shogaol (20, 40, 80 µM) had no or little cytotoxicity in normal human liver HL-7702 cells (Figure 1H).

### Cellular Proteins Alteration in Response to 6-shogaol Administration by 2-DE

**Figure 2:**
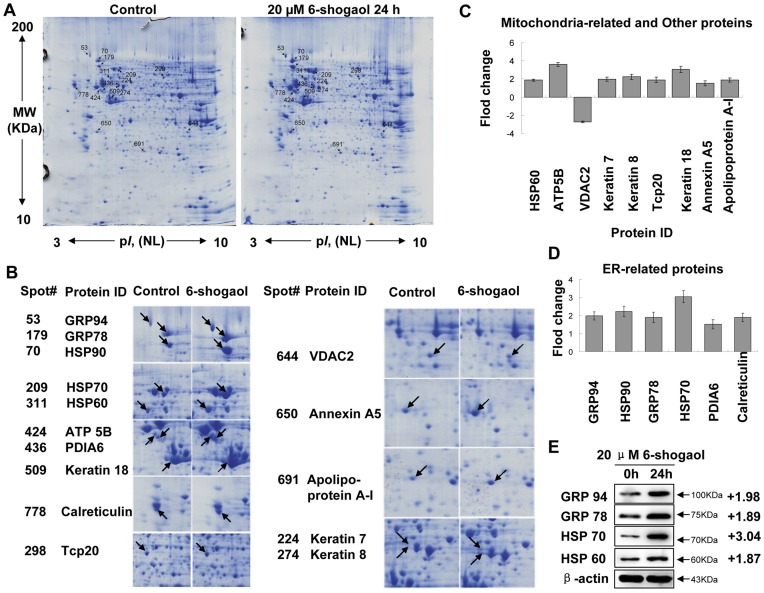
2-DE images of total cellular proteins extracted from 6-shogaol-treated SMMC-7721 cells. (A) SMMC-7721 cells were treated with or without 20 µM 6-shogaol for 24 h. Differentially expressed spots are shown by the arrows. (B) The expanded region of altered protein spots, the proteins with arrow are the differentially expressed proteins identified by MS. (C) The intensity of mitochondria-related and other proteins. (D) The intensity of ER-related proteins. (E) Western bolt imaging of GRP94, GRP78, HSP70 and HSP60. For Western blot analysis, each lane was loaded with 30 µg of protein. Blots were subsequently stripped and reprobed with antibody against β-actin to ensure equivalent loading and transfer. Two additional studies yielded equivalent results.

To further investigate the mechanism of 6-shogaol-induced apoptosis, protein profiles of control and 6-shogaol-treated cells were determined by 2-DE. 2-DE is one of the core technologies in proteomics used for separation and abundance analysis of proteins which are associated with cellular mechanisms. The alterations of cellular protein expression with drug treatment can provide valuable information to explore the molecular mechanism of drug actions [27]. Figure 2A shows the representative 2-DE gel images for total proteins extracted from SMMC-7721 cells treated without or with 20 µM 6-shogaol for 24 h. More than 900 protein spots were detected on the gel ranging from MW 10-200 kDa and p*I* 3–10. The significantly differentially expressed protein spots (up- or down-regulation over 1.5 fold) were selected for protein identification. Detailed protein alterations in expression were found as indicated by spots marked with arrows in Figure 2B. The differentially expressed proteins with their spot number, protein name, accession number, MW/p*I* values and scores are listed in Table 1. These altered proteins can be classified into three categories according to their main functions and locations in cells. The first group is located in the ER, which is related to protein synthesis and folding, including GRP78/Bip, GRP94, HSP90, Calreticulin, HSP70 and PDIA6, etc (Figure 2D). The second group is involved in energy production and mitochondrial translation, including ATP synthase subunit beta (ATP5B), VDAC2 and mitochondria chaperonins HSP60, etc (Figure 2C). Other altered proteins including up-regulated of keratin 7, keratin 8, keratin 18 and down-regulated of T-complex protein 1 (Tcp20) subunit zeta are located in cytoskeleton. In addition, calcium ion binding protein Annexin A5 and translation protein Apolipoprotein A-I were up-regulated (Figure 2C). Expression of GRP78, GRP94, HSP70 and HSP60 were verified by western blot (Figure 2E). The proteomic analysis by 2-DE revealed that ER stress-related proteins were significantly up-regulated in response to 6-shogaol treatment, suggesting that ER stress was involved in 6-shogaol induced apoptosis.

**Table 1:** Protein alterations in response to 6-shogaol treatment (20 µM for 24 h).

Spots	Protein name	Accession No.	MW(KDa)/*pI*	Protein score	Sequence coverage%	No. of Queries matched		Fold difference(6-shogoal: Ctrl)
**Endoplasmic reticulum**								
53	Endoplasmin	ENPL_HUMAN	92.7/4.76	392	24		23	1.98±0.23
70	Heat shock protein HSP 90-beta	HS90B_HUMAN	83.5/4.97	454	33		25	2.22±0.29
179	78 kDa glucose-regulated protein	GRP78_HUMAN	72.4/5.07	766	41		40	1.89±0.29
209	Heat shock 70 kDa protein1	HSP71_HUMAN	70.3/5.48	710	43		30	3.04±0.34
436	Protein disulfide-isomerase A6	PDIA6_HUMAN	48.6/4.95	166	22		7	1.52±0.26
778	Calreticulin (grp60)	CALR_HUMAN	48.3/4.29	197	30		10	1.89±0.23
**Mitochondria**								
311	60 kDa heat shock protein	CH60_HUMAN	61.2/5.7	610	45		36	1.87±0.13
424	ATP synthase subunit beta	ATPB_HUMAN	56.5/5.26	316	31		18	3.62±0.22
644	Voltage-dependent anion-selective channelprotein 2	VDAC2_HUMAN	32.1/7.49	139	27		6	–2.73±0.08
**Cytoplasm and Others**								
224	Keratin, type II cytoskeletal 7	K2C7_HUMAN	51.4/5.50	289	24		12	3.66±0.41
274	Keratin, type II cytoskeletal 8	K2C8_HUMAN	53.7/5.52	348	21		12	1.99±0.21
298	T-complex protein 1 subunit zeta	TCPZ_HUMAN	58.5/6.23	165	17		11	–3.59±0.31
509	Keratin, type I cytoskeletal 18	K1C18_HUMAN	48.0/5.34	160	31		15	1.75±0.19
650	Annexin A5	ANXA5_HUMAN	36.0/4.94	357	37		20	2.77±0.22
691	Apolipoprotein A-I	APOA1_HUMAN	30.7/5.56	87	15		3	1.65±0.12

### 6-shogaol Stimulated UPR and Induced Apoptosis through PERK/eIF2α Passway in SMMC-7721 Cells

**Figure 3:**
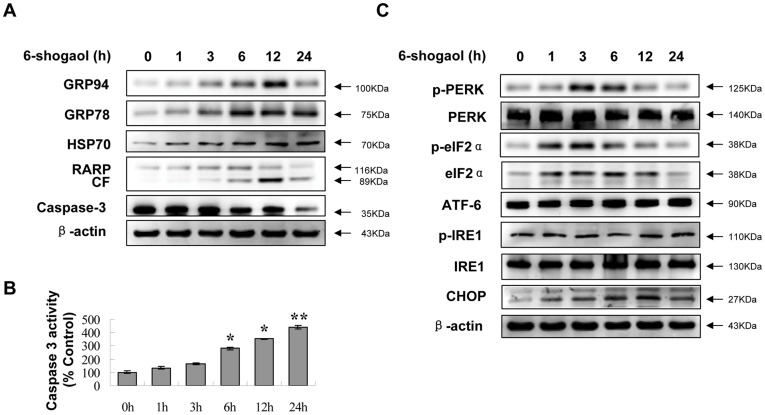
Effects of 6-shogaol on ER stress signaling proteins. The cells were treated with or without 20 µM 6-shogaol for 1, 3, 6, 12, and 24 h. Total cellular extracts were prepared and subjected to Western blot analysis using antibodies against PARP, caspase-3 and ER stress-related molecular chaperones (A) and ER stress related proteins (C). CF stands for cleavage fragments of PARP. The caspase-3 activity (B) was assayed according to Caspase-3 Activity Assay Kit. *Values for cells treated with 6-shogaol were significantly greater than the control group by Student’s *t*-test (*P*<0.05); **(*P*<0.01).

UPR is an important genomic response to ER stress. Time-dependent effects of 6-shogaol were examined in relation to UPR. As shown in Figure 3A, exposure of cells to 6-shogaol resulted in marked increase in expression of UPR targets Grp78/Bip, Grp94 and HSP70 in a time-dependent manner. Exposure of cells to 6-shogaol also resulted in PARP degradation and caspase-3 activation (Figure 3A and 3B). Interestingly, the levels of phospho-PERK, eIF2α and phospho-eIF2α were significantly increased at early time points (1, 3 and 6 h) (Figure 3C) but obvious apoptosis did not occur (Figure 1G). However, phospho-PERK and phospho-eIF2α were slightly decreased after 12 h, accompanied by increased apoptosis (Figure 1G and 3C). In contrast, 6-shogaol had little or no effect on the expression of other two UPR components IRE1 and ATF6 (Figure 3C). The present results suggest that PERK/eIF2α passway plays a vital role in 6-shogaol-induced apoptosis in SMMC-7721 cells, and apoptosis is augmented with the inhibition of the phosphorylation of eIF2α. It is reported that ER stress activates transcription factor C/EBP homologous protein (CHOP) and induces apoptosis through promoting ER client protein biosynthesis by dephosphorylating eIF2α [28]. In our investigation, the up-regulation of CHOP was observed in a time-dependent manner (Figure 3C).

### Salubrinal Synergizes with 6-shogaol to Induce Apoptosis in SMMC-7721 Cells

**Figure 4:**
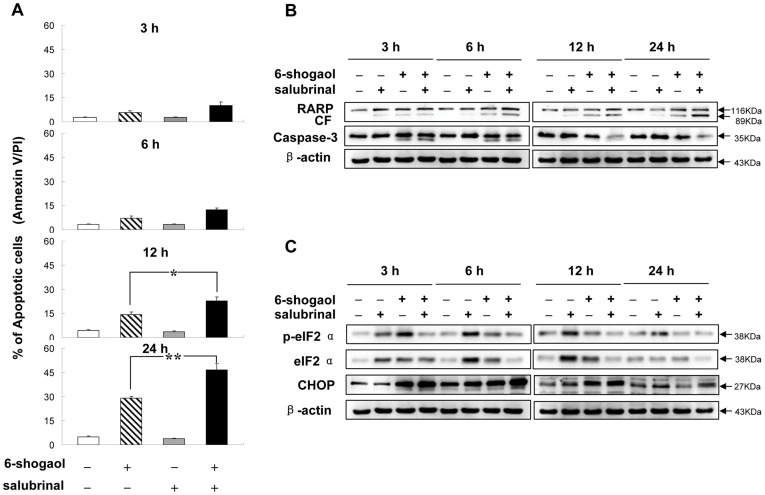
Effects of co-administration of salubrinal and 6-shogaol on apoptosis and phosphorylation of eIF2α. SMMC-7721 cells were pretreated with 5 µM salubrinal for 2 h, followed by treatment with 20 µM 6-shogaol for 3, 6, 12, and 24 h. (A) Apoptosis was determined using flow cytometry. *Values for cells treated with salubrinal and 6-shogaol in combination were significantly greater than those for cells treated with 6-shogaol alone by Student’s *t*-test; *P*<0.05. Total protein extracts were prepared and subjected to Western blot assay using antibodies against PARP and caspase-3 (B), and eIF2α pathway related proteins (C). CF stands for cleavage fragments of PARP.

Recently, several reports have demonstrated that eIF2α phosphorylation is required for cell survival. The absence of eIF2α phosphorylation enhances cell death [29], [30]. Salubrinal selectively blocks dephosphorylation of eIF2α and protects cells against ER stress-mediated apoptosis [20]. We therefore examined whether salubrinal protects SMMC-7721 cells against 6-shogaol-induced apoptosis. Unexpectedly, salubrinal potentiated the pro-apoptotic effect of 6-shogaol. As shown in Figure 4A, co-treatment with salubrinal and 6-shogaol for 3 and 6 h resulted in slight increase in apoptosis. Similarly, co-administration for 12 and 24 h resulted in significant increase in apoptosis (20.44±4.96% and 46.58±3.85%) compared to 6-shogaol treated alone (14.86±0.53% and 29.12±0.95%) (*p*<0.01, n = 3). Cytotoxicity of 6-shogaol enhanced by salubrinal was also reflected by degradation of PARP and activation of caspase-3 (Figure 4B). Taken together, these results suggest that salubrinal synergizes with 6-shogaol to induce apoptosis in SMMC-7721 cells.

### Salubrinal Synergizes with 6-shogaol to Attenuate eIF2α Phosphorylation and Up-Regulate CHOP Expression in SMMC-7721 Cells

We then investigated the role of salubrinal on eIF2α in SMMC-7721 cells. We performed a time course analysis of eIF2α phosphorylation in SMMC-7721 cells exposed to salubrinal and/or 6-shogaol. As shown in Figure 4C, Salubrinal increased the level of total eIF2α at 3 h, and reached near-maximal level at 12 h, then decreased to basic level at 24 h. Salubrinal alone increased eIF2α phosphorylation at different time points. And salubrinal treatment alone had no cytotoxic effect on SMMC-7721 cells. However, co-administration of salubrinal and 6-shogaol for different time intervals (3, 6, 12, and 24 h) resulted in marked inhibition of eIF2α phosphorylation. Meanwhile, a strong induction of the pro-apoptotic gene CHOP was observed. These results suggest that salubrinal significantly promoted apoptosis mediated by 6-shogaol through inhibition of eIF2α phosphorylation and induction of pro-apoptotic mediator CHOP in SMMC-7721 cells.

### Enforced Activation of eIF2α Potentiates eIF2α Phosphorylation and Attenuates 6-Shogaol-mediated Apoptosis

**Figure 5:**
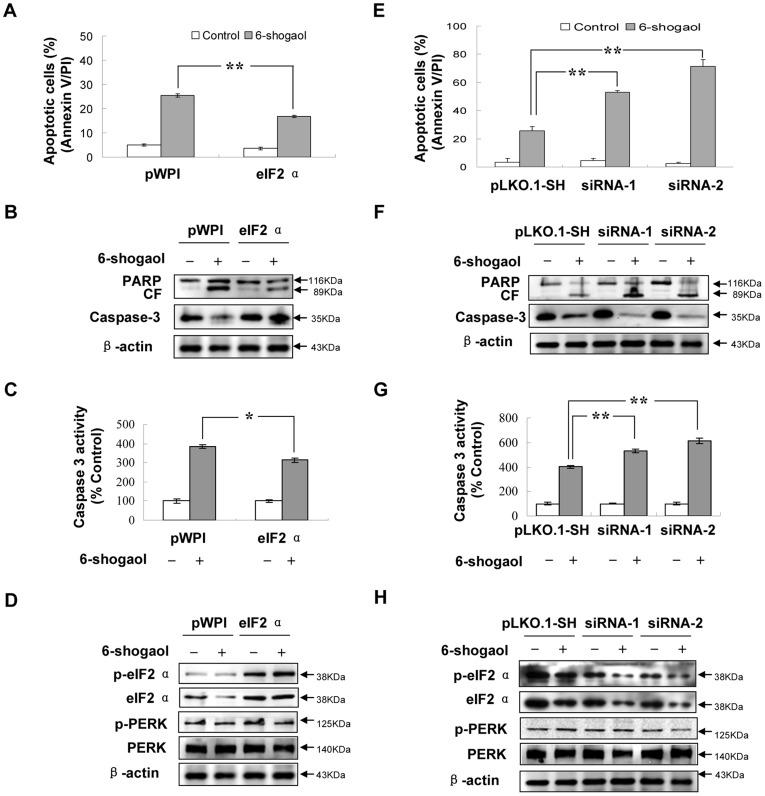
The role of eIF2α in 6-shogaol-induced apoptosis. SMMC-7721 cells were stably transfected with an empty vector (pWPI) or over-expression form of eIF2α as described in Materials and methods. All cells were then treated with 20 µM 6-shogaol for 24 h. (A) After treatment, apoptosis was determined using flow cytometry. **P<0.01. Total cellular extracts were prepared and subjected to (C) caspase-3 activity assay and Western blot assay using antibodies against PARP, caspase-3 (B) and PERK/eIF2α signaling proteins (D). SMMC-7721 cells were also stably transfected with an empty vector pLKO.1-SH or eIF2α siRNA (two clones siRNA-1 and siRNA-2). Cells were then treated with 20 µM 6-shogaol for 24 h. (E) After treatment, apoptosis was determined using flow cytometry. **P<0.01. Total cellular extracts were prepared and subjected to (G) caspase-3 activity assay and Western blot analysis using antibodies against PARP, caspase-3 (F) and PERK/eIF2α signaling proteins (H). CF stands for cleavage fragments of PARP.

To further elucidate the functional role of eIF2α in 6-shogaol-mediated lethality, SMMC-7721 cells over-expressing eIF2α were employed. As shown in Figure 5A, eIF2α over-expressing cells were markedly less sensitive to 6-shogaol-induced apoptosis than pWPI vector cells (*p*<0.01). Consistent with these findings, 6-shogaol was considerably less effective in triggering activation of caspase-3 and PARP degradation in eIF2α over-expressing cells compared to pWPI vector cells (Figure 5B and 5C). The levels of total eIF2α and phospho-eIF2α clearly increased in eIF2α over-expressing cells. There was hardly any inhibition of 6-shogaol induced eIF2α phosphorylation in these cells (Figure 5D). Together, these findings indicate that eIF2α may play a protective role against 6-shogaol-mediated lethality.

### Inhibition of eIF2α by siRNA Reduces eIF2α Phosphorylation and Enhances 6-shogaol-Mediated Apoptosis

In order to investigate the role eIF2α played in 6-shogaol-mediated apoptosis, a genetic approach utilizing eIF2α siRNA (siRNA-1 and siRNA-2) was also employed. Transfection of SMMC-7721 cells with eIF2α siRNA reduced expression of eIF2α apparently. Accordingly, phosphorylation of eIF2α in cells transfected with eIF2α siRNA was substantially reduced (Figure 5H). As shown in Figure 5E, SMMC-7721 cells transfected with eIF2α siRNA were significantly more sensitive to 6-shogaol-mediated lethality than pLKO.1-SH control vector treated cells (*p*<0.01). Consistent with these results, 6-shogaol was considerably more effective in triggering activation of caspase-3 and PARP degradation in eIF2α siRNA cells (Figures 5F and 5G). Taken together, these findings indicate that inhibition of eIF2α phosphorylation enhances apoptosis in 6-shogaol treated SMMC-7721 cells.

### 6-Shogaol Exhibits Antitumor Activity in SMMC-7721 Xenografts

**Figure 6:**
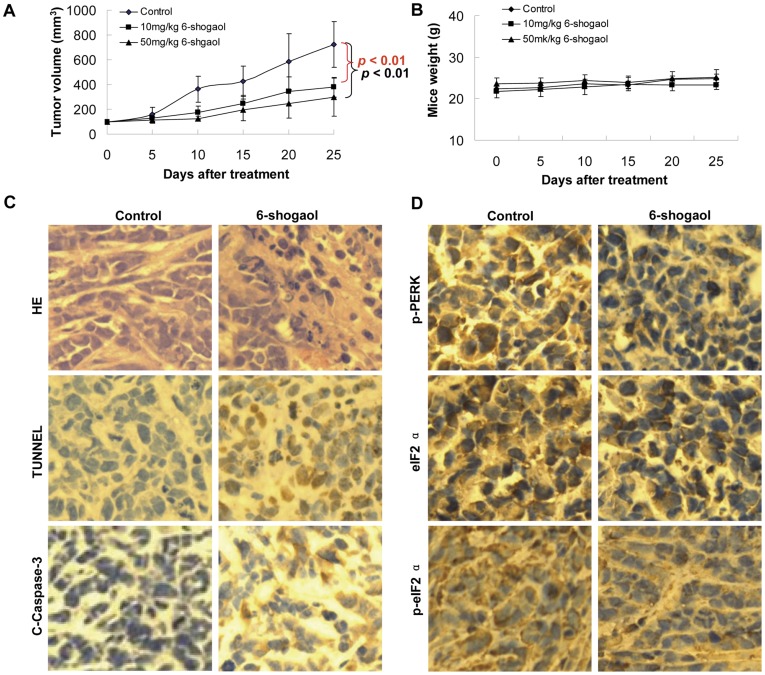
*In vivo* antitumor effects of 6-shogaol in SMMC-7721 xenografts. SCID mice were inoculated with SMMC-7721 cells (3×10^6^ cells/mouse, s.c.) and randomly divided into three groups (8/group) treated with 6-shogaol (10 mg/kg and 50 mg/kg, i.p., daily) or vehicle control solvent. (A) Average tumor volume in vehicle control mice and mice treated with 10 mg/kg and 50 mg/kg 6-shogaol. ** *P*<0.01. (B) Body weight changes of mice during the 28 days of study. (C) Representative photographs of biopsy samples from mice treated with 6-shogaol (50 mg/kg) and vehicle control. Original magnification ×400. (D) Expression of p-PERK, eIF2α, and p-eIF2α in tumor tissues of SMMC-7721 xenograft mouse model treated with 6-shogaol (50 mg/kg) and vehicle control.

To verify the therapeutic effect of 6-shogaol, we further applied 6-shogaol to SMMC-7721 xenografts to evaluate its significance *in vivo*. As shown in Figure 6A, treatment with 6-shogaol significantly inhibited tumor growth. The mean volume of tumors in mice treated with 6-shogaol (10 mg/kg and 50 mg/kg) was much smaller than the tumors in the vehicle-control mice (*p*<0.01). There is no significant difference in body weight between 6-shogaol treatment and vehicle-control group (Figure 6B), indicating that no severe toxicity was observed.

To evaluate whether administration of 6-shogaol induces apoptosis in SMMC-7721 cells *in vivo*, tumor sections from vehicle and 6-shogaol-treated mice were stained with H&E or TUNEL. As shown in Figure 6C (top panel), the sections of SMMC-7721 xenografts from mice treated with 6-shogaol showed that cancer cells markedly decreased, with signs of necrosis with infiltration of inflammatory cells, fibrosis, as well as apoptotic regions, identified by their amorphous shape and condensed nuclei. Feature of apoptosis were also observed in the tumor sections stained with TUNEL. The sections of tumor from 6-shogaol-treated mice showed numerous dark brown colored apoptotic cells (Figure 6C, middle panel).

To further correlate the anti-tumor molecular mechanism of 6-shogaol *in vitro*, tumor sections from vehicle and 6-shogaol-treated mice were investigated by immunohistochemistry analysis to detect clevead-caspase-3, phospho-PERK, eIF2α and phospho-eIF2α. As expected, phospho-PERK, eIF2α and phospho-eIF2α (Figure 6D) decreased, and cleveage-caspase-3 increased in 6-shogaol-treated tumor (Figure 6C bottom panel).

Overall, these findings demonstrate that 6-shogaol administration significantly inhibites SMMC-7721 xenograft growth without causing any side effects to the mice. 6-shogaol-mediated antitumor activity *in vivo* is associated with induction of apoptosis and inhibition of eIF2α phosphorylation.

## Discussion

In the present study, we demonstrated that 6-shogaol induced apoptosis in SMMC-7721 cells and showed very low cytotoxicity in normal liver HL-7702 cells. The possible cellular mechanism of 6-shogaol-induced apoptosis was investigated. 6-Shogaol could stimulate ER stress in SMMC-7721 cells. Exposed to 6-shogaol for a long time could induce prolonged ER stress. Dephosphorylation of PERK/eIF2α and activate the downstream CHOP expression triggered caspase cascade reaction to induce apoptosis in SMMC-7721 cells.

The proteomic analysis revealed that ER molecular chaperones were significantly up-regulated in response to 6-shogaol treatment, suggesting that ER stress was induced by 6-shogaol. UPR is an important genomic response to ER stress and is characterized by the activation of three distinct signal transduction pathways mediated by PERK, IRE1 and ATF6. Our findings here demonstrated that 6-shogaol triggered UPR and selectively activated PERK/eIF2α pathway at the early stage. At this stage, apoptosis was not obvious. However, long time 6-shogaol treatment induced inhibition of eIF2α phosphorylation associated with increased apoptosis. It is suggested that eIF2α phosphorylation is a key step in maintaining a balance between survive and death of 6-shogaol-treated SMMC-7721 cells. eIF2α phosphorylation shifts from a cyto-protective to a pro-apoptotic state in response to sustained 6-shogaol treatment.

Salubrinal was reported to increase eIF2α phosphorylation and protect cells against ER stress-mediated apoptosis [20], [31], [32]. In our investigation, salubrinal treatment alone selectively blocked dephosphorylation of eIF2α and did not affect SMMC-7721 cells apoptosis. However, salubrinal specifically potentiated 6-shogaol-induced apoptosis in SMMC-7721 cells by inhibition of eIF2α phosphorylation and induction of the pro-apoptotic gene CHOP. It can be concluded that in SMMC-7721 cells, increased eIF2α phosphorylation by salubrinal encouraged the ER stress induced by 6-shogaol, so the excessive ER stress could be achieved in advance. Apoptosis was triggered at an early stage. The cellular mechanism of synergetic apoptotic effect of salubrinal and 6-shogaol needs to be further investigated. However, it was confirmed that inhibition of eIF2α phosphorylation and increased expression of CHOP enhanced the lethality of 6-shogaol in SMMC-7721 cells. Clearly, over-expression of eIF2α, and induction of eIF2α phosphorylation, largely reversed the lethal consequences of 6-shogaol exposure, including caspase activation, PARP cleavage, and apoptosis. On the other hand, genetic interruption of eIF2α with siRNA, which substantially diminished eIF2α phosphorylation, potentiated 6-shogaol-mediated lethality. It is demonstrated that eIF2α phosphorylation is required for cell survival, the absence of eIF2α phosphorylation enhances cell death.

Recent studies have shown that 6-shogaol is a strong inducer of apoptosis in diverse cancer cells [4]-[8]. However, it is unclear whether 6-shogaol-induced apoptosis of HCC cells *in vivo* occurs in response to treatment with this compound *in vitro*. In the present study, we have shown that induction of apoptosis by 6-shogaol indeed occurred *in vivo*, which could be responsible for the inhibitory effects on tumor growth by 6-shogaol. Consistent with the *in vitro* data, our *in vivo* results indicate that suppression of phospho-PERK, phospho-eIF2α, and eIF2α is closely correlated with the reduction of SMMC-7721 tumor xenografts, further supporting our notion.

In conclusion, the present findings indicate that 6-shogaol induces apoptosis, caspase activation, and PARP cleavage in both SMMC-7721 cells and SMMC-7721 tumor xenografts through an ER stress-associated mechanism. Our data indicate that attenuation of eIF2α phosphorylation and up-regulation of CHOP may contribute to 6-shogaol-mediated apoptosis in SMMC-7721 cells. Because 6-shogaol selectively kills HCC cells without a significant toxic effect on normal liver cells and had little toxicity in SMMC-7721 xenograft mice, it seems that 6-shogaol may have clinical implications for therapeutic intervention against HCC or other malignancies. Our findings also show that salubrinal enhances 6-shogaol induced apoptosis through inhibition of eIF2α phosphorylation. The combination of 6-shogaol and salubrinal treatment may be considered as a novel therapeutic strategy in HCC.

## Supporting Information

Figure S1 **Effect of 6-shogaol on apoptosis in SMMC-7721 cells.** (A) Cells (1×10^6^) were treated with 20 µM 6-shogaol for 6, 12, 24 h and stained with PI for flow cytometry assay (BD FACS Calibur) 20,000 cells were analyzed using the DNA analysis software (ModFitLT V3.2). (B) SMMC-7721 cells were treated without or with 20 µM 6-shogaol for 3, 6, 12, 24 h. Total cellular extracts were prepared and subjected to Western blot analysis using antibodies against calpain-1 and calpain-2. (C) SMMC-7721 cells were treated with 20 µM 6-shogaol for 3, 6, 12, 24 and 48 h. The cells were stained with Annexin V/PI, and apoptosis was determined using flow cytometry [25]. (D) Cells (1×10^6^) were treated with 20 µM 6-shogaol for 3, 6, 12, 24 h and stained with Hoechst 33258. Original magnification ×400.
(TIF)
