# Supplementary figures and images for: 6-Shogaol Induces Apoptosis in Human Hepatocellular Carcinoma Cells and Exhibits Anti-Tumor Activity In Vivo through Endoplasmic Reticulum Stress
Source: PLoS One. 2012 Jun 29;7(6):e39664. doi: 10.1371/journal.pone.0039664 (PMC3387266; doi:10.1371/journal.pone.0039664)

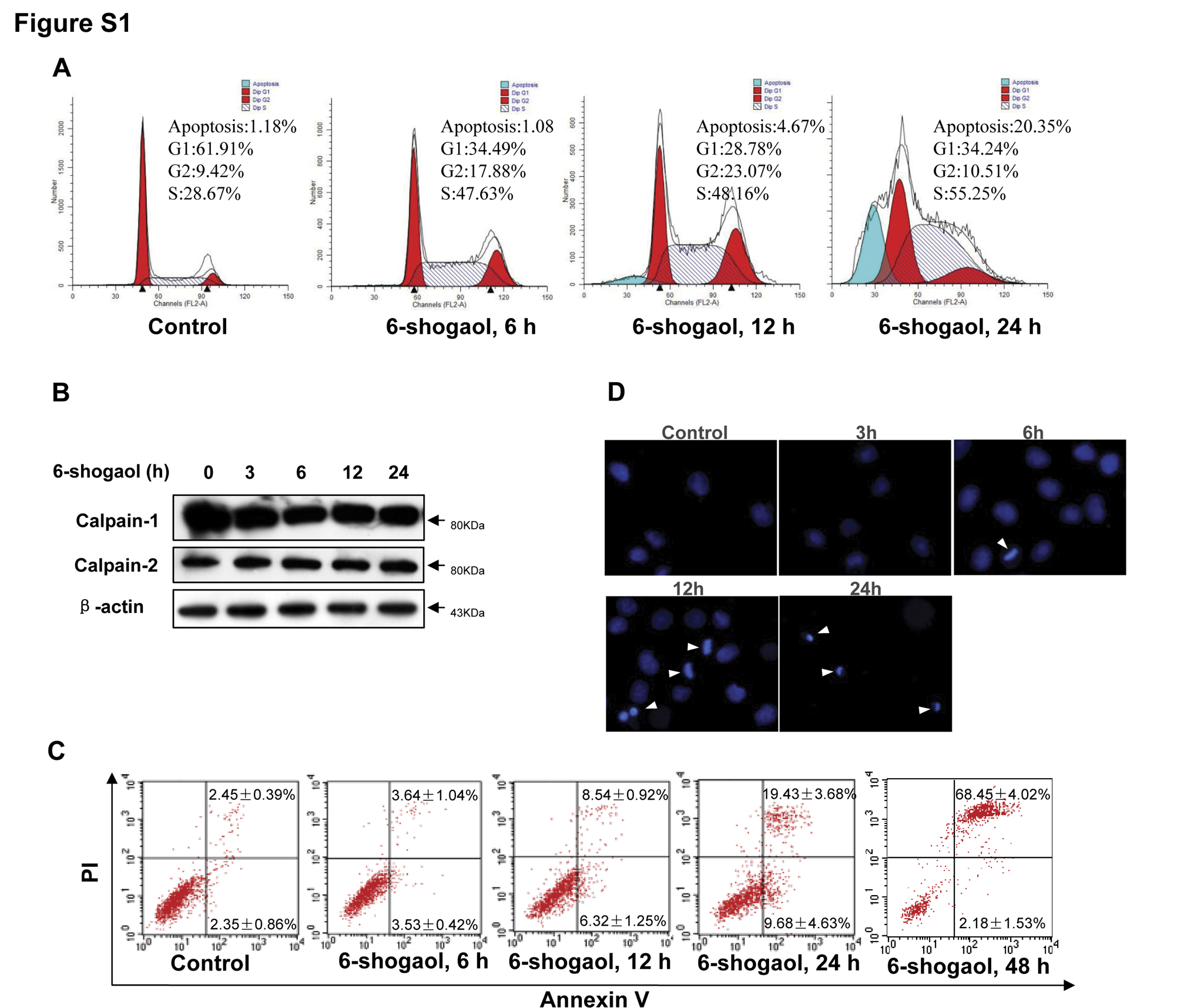

Supplement: Figure S1 — Effect of 6-shogaol on apoptosis in SMMC-7721 cells. (A) Cells (1×10^6^) were treated with 20 µM 6-shogaol for 6, 12, 24 h and stained with PI for flow cytometry assay (BD FACS Calibur) 20,000 cells were analyzed using the DNA analysis software (ModFitLT V3.2). (B) SMMC-7721 cells were treated without or with 20 µM 6-shogaol for 3, 6, 12, 24 h. Total cellular extracts were prepared and subjected to Western blot analysis using antibodies against calpain-1 and calpain-2. (C) SMMC-7721 cells were treated with 20 µM 6-shogaol for 3, 6, 12, 24 and 48 h. The cells were stained with Annexin V/PI, and apoptosis was determined using flow cytometry [25]. (D) Cells (1×10^6^) were treated with 20 µM 6-shogaol for 3, 6, 12, 24 h and stained with Hoechst 33258. Original magnification ×400. (TIF) [file pone.0039664.s001.tif]
